# *Akkermansia* beyond muciniphila - emergence of new species *Akkermansia massiliensis* sp. nov.

**DOI:** 10.20517/mrr.2024.28

**Published:** 2024-06-25

**Authors:** Ritesh Kumar, Oliver Hasselwander, Helene Kane, Ashley A Hibberd

**Affiliations:** ^1^Health & Biosciences, International Flavors & Fragrances, Inc. (IFF), Wilmington, DE 19803, USA.; ^2^Health & Biosciences, IFF, c/o Danisco UK Ltd., Reigate RH2 9PW, UK.; ^3^Health & Biosciences, IFF, Saint Louis, MO 63110, USA.

**Keywords:** *Akkermansia muciniphila*, *Akkermansia massiliensis* sp. nov., *Akkermansia biwaensis* cardiometabolic health

## Abstract

The human gut commensal *Akkermansia muciniphila* is the most studied bacterial species within the Verrucomicrobiota phylum. It has been proposed as a beneficial next-generation probiotic for cardiometabolic and immune health. Efforts from various research groups have resulted in the recent discovery of new species and/or phylotypes of the genus *Akkermansia*. This highlighted the genetic and phenotypic diversity among the *Akkermansia* isolates, providing an opportunity to identify novel mechanisms pertaining to health benefits. Genetic diversity between strains warrants detailed investigation to determine beneficial *Akkermansia* strains. *Akkermansia massiliensis* sp. nov. has emerged as the second most prevalent *Akkermansia* species in the human gut, with unique properties and potential relevance for human health. In addition, indications of the co-existence of more than one *Akkermansia* phylotype or species in a subject are intriguing. These new discoveries pave the way for additional developments of effective and targeted *Akkermansia* species-based interventions to provide health benefits.

## BACTERIAL SPECIES OF THE GENUS *AKKERMANSIA*

The genus *Akkermansia* belonging to the phylum Verrucomicrobiota of Gram-negative bacteria was first described by scientists at the University of Wageningen, Netherlands, after isolation and characterization of a mucin-degrading strain from a healthy adult, which they named *Akkermansia muciniphila* and is represented by the type strain MucT (= ATCC BAA-835)^[1]^. Since then, two more validly published species under The International Code of Nomenclature of Prokaryotes (ICNP) of the genus *Akkermansia* have been identified: *A. glycaniphila* isolated from reticulated python feces (PytT)^[2]^ and *A. biwaensis* (type strain WON2089T)^[3]^.

**Figure 1:**
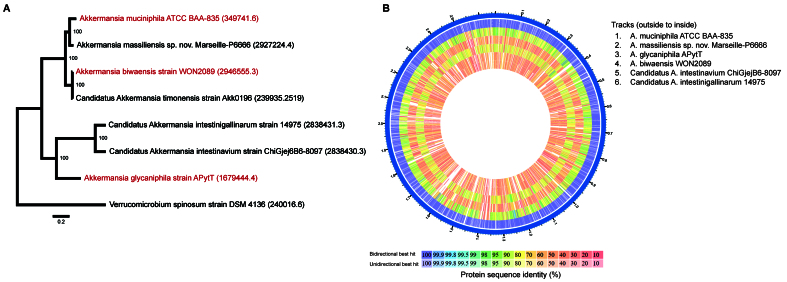
Genomic phylogenetic tree and corresponding proteome^[6]^. Type strains of validly published species are denoted in red.

In addition, several new *Akkermansia* species have been proposed, such as *Candidatus A*. *intestinavium* isolated from the gut of birds^[4]^, *Candidatus A. intestinigallinarum* isolated from the gut of hens^[4]^, and the human gut isolates *A. massiliensis* sp. nov. with strain Marseille-P6666T (= CSUR P6666 = CECT 30548) as the type strain^[5]^ and *Candidatus A. timonensis* with Akk0196 as the type strain^[5]^. Figure 1 shows the genomic phylogenetic tree and corresponding proteome of the current and proposed *Akkermansia* species generated using the BV-BRC server^[6]^. Interestingly, the recently named *A. biwaensis* and the proposed *Candidatus A. timonensis* type strains are 99.5% similar based on genome-wide average nucleotide identity (gANI); hence, they represent the same species. *A. biwaensis* is on the notification list for taxonomic validation^[7]^. A recent phylogenomic analysis of 367 high-quality *Akkermansia* isolates and metagenome-assembled genomes suggested the existence of at least 25 *Akkermansia* species at the genomic level^[8]^.

Based on extensive metagenomic assembly, *Akkermansia* strains in the human gut were grouped into five candidate species including *A. muciniphila* based upon species-level genome bins (SGBs), with significant genomic diversity despite similar 16S rRNA gene sequences^[9]^. Karcher *et al.* highlighted that this surprisingly high similarity based on 16S rRNA (at least 98%) is likely the reason for the wrong taxonomic assignment of several cultivated strains to the *A. muciniphila* species and leading to an underestimated diversity of the genus *Akkermansia* and less researched and characterized *Akkermansia* species beyond *A. muciniphila*^[9]^. The publication also highlighted that the corrin ring biosynthesis operon genes are consistently present only in two candidate species, namely SGB9227 and SGB9228, but not *A. muciniphila*. A comparison of the public genome of cultured isolates from NCBI within SGB9228 to the proposed type strain for *A. massiliensis* sp. nov. Marseille-P6666T shows that SGB9228 corresponds to the proposed *A. massiliensis* species (> 95% gANI similarity), and SGB9224 similarly corresponds to *A. biwaensis*.

## *AKKERMANSIA MASSILIENSIS* SP. NOV. *-* A RE-EXAMINATION OF ITS PRESENCE IN THE GUT MICROBIOME

In the survey published by Karcher *et al*.^[9]^, they reported the prevalence of *A. muciniphila* in gut metagenomes from adults (*n* = 7,995) as 34%, *A. massiliensis* sp. nov. (SGB9228) as 8%, and *A. biwaensis* (SGB9224) as 2%. While it is true that the high similarity of the 16S rRNA genes among these species and the lack of reference sequences in taxonomic databases contributed to their historical under-classification in microbiome datasets, these species can be distinguished using newer high-resolution clustering algorithms such as DADA2 that defines amplicon sequence variants (ASVs) based on single base pair differences^[10]^. If utilizing the commonly sequenced V4 region of the 16S rRNA gene (515f/806r primer pair), there are four base pair differences between each *A. muciniphila* (GenBank Accession: AY271254) and *A. massiliensis* sp. nov. (GenBank Accession: ON014381) and likewise between *A. muciniphila* and *A. biwaensis* (GenBank Accession: LC711105)*,* which provides sufficient resolution to distinguish ASVs from the species within *Akkermansia*. Thus, we examined the prevalence and abundance of these species in three studies where fecal 16S rRNA V4 amplicon data were collected: (1) The American Gut Project^[11]^ (*n* = 5,000), which involved a crowd-sourced sampling of adults; (2) IsoMic clinical study (ClinicalTrials.gov identifier NCT05150184), which was comprised of healthy adults [Body mass index (BMI) 18.5-25 kg/m^2^] from 5 ethnicities residing in the United States; and (3) Next Generation Probiotics for Metabolic Health (NGPs for MH) clinical study (ClinicalTrials.gov identifier NCT04229082), which included lean (BMI 18-25 kg/m^2^) and obese (BMI 27.5-35 kg/m^2^) women residing in Finland. Similar to Karcher *et al*.^[9]^, we found *A. muciniphila* to be both more prevalent and abundant in all of the cohorts compared to *A. massiliensis* sp. nov. [Table 1]. The prevalence of *A. massiliensis* sp. nov. was higher in the American Gut Project, IsoMic, and NGPs for MH lean cohorts (14.7%, 11.5%, and 26.3%, respectively), compared to the average abundance (8%) reported by Karcher *et al.*^[9]^. The exception was the NGPs for MH obese cohort, where, intriguingly, the colonization of *A. massiliensis* sp. nov. was not detected in any of the individuals. Interestingly, only a small percentage of individuals were co-colonized with both *A. municiphila* and *A. massiliensis* sp. nov., suggesting that these two species may be mutually exclusive. This agrees with Karcher *et al*.^[9]^, who also showed a strong negative correlation between their co-occurrence in 4,171 human metagenomes. *A. biwaensis* was undoubtedly the most rarely detected human-associated *Akkermansia* species, but we found its prevalence was 8.9% in the American Gut Project cohort, with an average abundance of 0.2%. This prevalence is perhaps higher than expected for this almost completely unexplored species in human microbiomes. These findings support the exploration of potential metabolic health benefits associated with the colonization of *A. massiliensis* sp. nov. and warrant future mechanistic studies to better understand the interplay among *A. muciniphila* and the less abundant *Akkermansia* species and their impact on the host.

**Table 1:** Prevalence and abundance of *Akkermansia muciniphila* and *Akkermansia massiliensis* sp. nov. detected by 16S rRNA amplicon sequencing in study cohorts

**Study**	**Cohort**	***n***	***Akkermansia muciniphila* ^1^**		***Akkermansia massiliensis* sp. nov.^2^**		**Co-colonized^3^**
			**Prevalence (%)**	**Average abundance (%)**	**Prevalence (%)**	**Average abundance (%)**	**Prevalence (%)**
American gut^[11]^	Crowd-sourced adult	5,000	47.5	1.0	14.7	0.3	1.3
IsoMic (NCT05150184)	Healthy adult	52	26.9	0.4	11.5	0.1	1.9
NGPs for MH (NCT04229082)	Lean adult	57	87.7	1.5	26.3	0.3	17.5
NGPs for MH (NCT04229082)	Obese adult	39	82.1	2.0	0.0	0.0	0.0

1 Sequence matching V4 region of 16S rRNA of *Akkermansia muciniphila* strain BAA-835T. ^2^Sequence matching V4 region of 16S rRNA of *Akkermansia massiliensis* sp. nov. strain Marseille-P6666T. ^3^Presence of both *Akkermansia muciniphila* and *Akkermansia massiliensis* sp. nov. in the same individual. NGPs for MH: Next-generation probiotics for metabolic health.

Two studies have looked in more detail at associations between colonization with different *Akkermansia* species and respective health outcomes. Karcher *et al.* reported that in a sample set of 3,311 genomes coming from different metagenomic datasets and isolates, only *A. muciniphila* was significantly negatively associated with BMI^[9]^. In addition, a more recent analysis by Mueller *et al.*, who re-analyzed metagenomic datasets of patients with obesity, inflammatory bowel disease, and response to cancer immunotherapy, reported that there were no significant correlations between abundances of *A. muciniphila* and *A. massiliensis* and obesity in children^[12]^. Only *A. muciniphila* was, however, found to be significantly reduced in samples from patients with ulcerative colitis compared to healthy controls. In addition, patients with non-small-cell lung cancer undergoing immune checkpoint inhibitor treatment colonized with *A. muciniphila* clade AmIa had an increase in survival compared to patients with no detectable *A. muciniphila*. Mueller *et al.* reported the prevalence of the different *Akkermansia* species similar to those in Table 1, with *A. muciniphila* being the most prevalent species [32.5% of 1,088 samples, followed by *A. massiliensis* (11.9%) and *A. biwaensis* (5.2%)]^[12]^. This lower prevalence of *Akkermansia* species other than *A. muciniphila* means that much larger datasets are required to thoroughly investigate correlations between human health outcomes and the relative abundance of these *Akkermansia* species.

At present, there is little known about the possible health effects of *Akkermansia* species other than *A. muciniphila*. However, Kumar *et al.* have first described the identification and characterization of a novel species of the genus *Akkermansia* (*Akkermansia* strain DSM 33459) with beneficial effects in relation to metabolic health effects demonstrated in a diet-induced obesity (DIO) mouse model^[13]^. The genome of this *Akkermansia* strain DSM 33459 showed 99.85% gANI similarity to the genome of the type strain *A. massiliensis* sp. nov. Marseille-P6666, indicating that strain DSM 33459 also belongs to the proposed species *A. massiliensis* sp. nov. and not to *A. muciniphila* species based on only 87.5% gANI identity. In the DIO model, *Akkermansia* DSM 33459 administration showed significant improvements in body weight, total fat weight, insulin and resistin levels after administration for 12 weeks^[13]^. Mice consuming the high-fat diet also harbored a significantly greater abundance of native *A. muciniphila* in their intestinal microbiota compared to mice on normal chow. *Akkermansia* DSM 33459 was able to efficiently engraft and replace the native *A. muciniphila* in this model. This further highlights the possible competition of the two *Akkermansia* species, which may limit their co-existence in the gut and questions what mechanisms may be at play, such as potential differences in their growth rates, their ability to adhere to intestinal epithelial cells, and metabolic pathways related to sulfur reduction^[14]^. Interestingly, in this mouse DIO study, pasteurized *A. massiliensis* sp. nov. did not show the same efficacy as compared to the live bacterium. This observation is different compared to what is known for *A. muciniphila*. Further studies are needed to explore the impact of pasteurization methods on the bioactivity of pasteurized *A. massiliensis* sp. nov.

In addition, van der Lelie *et al*. described a consortium of 11 strains (GUT-108), which was designed to address gut microbiome dysbiosis in inflammatory bowel disease, and showed that this consortium reversed experimental colitis in a mouse model^[15]^. One of the 11 strains was named *Akkermansia* sp. GGCC_0220 and was suggested to belong to a new *Akkermansia* species based on gANI. Our analysis of the public whole genome deposit record (GenBank Accession: JABFCI000000000.1) for this bacterium indicates that *Akkermansia* GGCC_0220 is 98.7% similar to both *A. massiliensis* sp. nov. Marseille-P6666 and *A. massiliensis* sp. nov. DSM 33459, i.e., it belongs to the same species.

## *AKKERMANSIA MASSILIENSIS* SP. NOV. - CHARACTERISTICS WITH RELEVANCE FOR UNIQUE MECHANISM OF ACTION

The human gut resident *A. muciniphila* has gained attention in recent years due to its potential role in promoting health. While research on *Akkermansia* is still progressing, several studies have suggested beneficial effects on health, including positive impacts on cardiometabolic health, inflammation, gut barrier integrity, outcomes to checkpoint blockade response in cancer immunotherapy, homeostatic immunity, and maintenance of microbial balance in the gut^[16,17]^. Research on *Akkermansia* is moving beyond association studies, with research increasingly focusing on the elucidation of the mechanisms underlying efficacy. Several possible mechanisms have been published to demonstrate the dynamics of this commensal in the gut. Plovier *et al.* have shown very elegantly the role of an outer membrane protein Amuc_1100 in metabolic and gut health^[18]^. Yoon *et al.* have identified another factor, P9 protein encoded by Amuc_1631, which interacts with ICAM2 and stimulates GLP1 secretion^[19]^. Recently, it has been shown that mediated production of a tripeptide [Arg-Lys-His (RKH)] can negate inflammation via TLR4 in a sepsis model^[20]^. Bae *et al.* have identified an active lipid molecule from the *A. muciniphila* membrane and the immunogenic activity of diacyl phosphatidylethanolamine with two branched chains (a15:0-i15:0 PE) has been shown to be via an unexpected toll-like receptor TLR2-TLR1 heterodimer^[21]^. Interestingly, Kumar *et al.* have shown variation in a15:0-i15:0 PE between *A. muciniphila* and *A. massiliensis* sp. nov., where *A. massiliensis* sp. nov. shows a higher abundance of a15:0-i15:0 PE as compared to *A. muciniphila*^[13]^. It will be worth investigating the impact of differential abundance of a15:0-i15:0 PE on the immunogenic properties of different *Akkermansia* strains.

Bacterial-derived extracellular vesicles have been investigated for their impact on physiological functions. Chelakkot *et al.* investigated the role of *A. muciniphila*-derived extracellular vesicles (AmEV) and showed that AmEV administration enhanced tight junction function, reduced body weight gain, and improved glucose tolerance in high-fat diet-induced diabetic mice^[22]^. What is unknown at present is whether AmEV serve as carriers for any of the possible effector molecules such as a15:0-i15:0 PE, Amuc_1100, and P9 or they act via another mechanism to provide the described benefits.

Advances in sequencing and bioinformatic tools have resulted in the identification of new *Akkermansia* phylotypes or species and highlighted the genomic and phenotypic diversity in the genus *Akkermansia*. The actual isolation of such novel *Akkermansia* strains enables further exploration in the field with the objective to understand if this variation is associated with unique properties and health benefits of the different *Akkermansia* species and phylotypes. This diversity also provides the opportunity to identify new mechanisms of action to understand differences in interaction between *Akkermansia* species and the host, raising interesting questions about the relative expression of effector molecules between different species and how this may impact efficacy. Recently, Kumar *et al.* have shown the health benefits of *A. massiliensis* sp. nov. in a DIO mouse model and have highlighted several new possible mechanisms of action^[13]^. They have shown that *A. massiliensis* sp. nov. can degrade extracellular adenosine triphosphate (eATP). It has been shown that eATP is a key inducer of inflammation in the gastrointestinal tract and is strongly associated with inflammatory bowel disease^[23]^. Extracellular ATP limits T follicular helper cells in the Peyer’s patches and thereby limits SIgA generation*;* thus*,* the degradation of eATP mediated by *Akkermansia* sp. in the lumen may play a vital role in modulating the immune structure in the gut^[24]^. Kumar *et al.* also described microbial agmatine production by the *A. massiliensis* sp. nov. strain, and agmatine has been shown to have metabolic and neuroprotective effects^[13,25]^. The *in vitro* production of agmatine was higher with *A. massiliensis* sp. nov. compared to *A. muciniphila* based on relative area concentrations. Pryor *et al.* have demonstrated that microbial agmatine is a major contributor to the agmatine pool in the gastrointestinal tract^[25]^. If it can be shown that *Akkermansia* contributes to this pool, then exploring the role of *Akkermansia* in relation to the gut-brain axis presents an interesting avenue to explore. Further *in vivo* studies are needed to validate the physiological significance of eATP degradation and agmatine production by *Akkermansia* sp.

**Figure 2:**
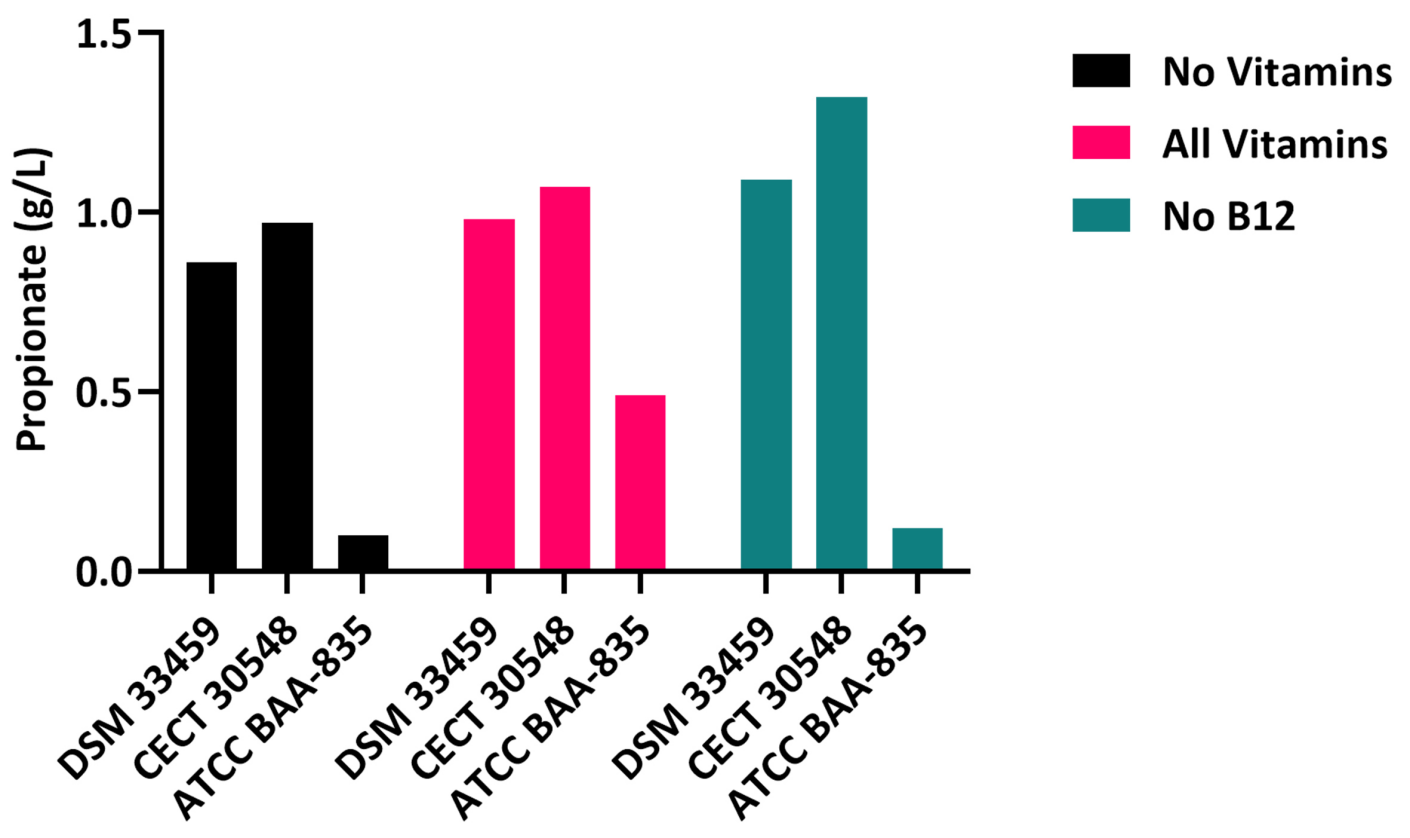
Functional characterization of propionate synthesis pathway of *Akkermansia* species using representative isolates: *Akkermansia massiliensis* sp. nov strain DSM 33459, *A. massiliensis* strain Marseille-P6666T (CECT 30548), and *Akkermansia muciniphila* strain BAA-835T (ATCC BAA-835).

Karcher *et al.* showed that the absence of the corrin ring biosynthesis operon genes in *A. muciniphila* resulted in an inability to produce propionate in the absence of vitamin B_12_^[9]^. As depicted in Figure 2, we similarly measured the Vitamin B_12_-dependent production of propionate in strains *Akkermansia massiliensis* sp. nov. DSM33459, Type *Akkermansia massiliensis* CECT 30548 and Type *Akkermansia muciniphila* ATCC BAA-835. Cells were grown in the defined media as published by Karcher *et al.*^[9]^. Supernatants were collected at 48 h of growth and propionate was measured using HPLC. Strains *A. massiliensis* sp. nov. DSM33459 and Type *A. massiliensis* sp. nov. CECT 30548 showed similar production of propionate irrespective of the addition of B_12_ in the media. Type *A. muciniphila* ATCC BAA-835 strain showed propionate production only in the presence of externally added B_12_, suggesting its dependency on the external B_12_ for propionate production as shown by Kirmiz *et al.* and Karcher *et al.*^[9,26]^. Propionate production is dependent on the B_12_-dependent methyl-malonyl CoA synthase reaction^[27]^. The presence of the corrin ring biosynthesis operon and, hence, the ability to synthesize vitamin B_12_ in *A. massiliensis* sp. nov. broadens its potential for human health. Recently, it has been shown that gut resident vitamin B_12_-producing bacteria can modulate excitatory cholinergic signaling and behavior in the host *Caenorhabditis elegans*^[28]^*.* Additionally, it is currently unknown, but possible that *Akkermansia* sp. may play a role in shaping the microbiome in the human gastrointestinal tract by providing the enzyme co-factor vitamin B_12_ to other bacterial species.

While these newly proposed mechanisms require further investigation and confirmation in *in vivo* studies, they do support further investigation of *A. massiliensis* sp. nov. in first-in-human intervention studies to study possible health benefits. The human gut commensal *A. muciniphila* remains the most studied beneficial bacterial species within the genus *Akkermansia*; however, the emergence of new species such as *A. massiliensis* sp. nov. presents new opportunities for the development of effective and targeted *Akkermansia* species-based interventions to provide health benefits.

## DECLARATIONS

### Copyright

© The Author(s) 2024.
